# The Role of Five Key Minerals (Cu, Se, Zn, Co, Fe) in Reproductive Function of Female Cattle: Current Insights and Future Directions

**DOI:** 10.3390/vetsci13020208

**Published:** 2026-02-23

**Authors:** Beiyao Wang, Xinlin Li, Zimo Zhou, Yanqiu Zhu, Zhicai Zuo, Hongrui Guo

**Affiliations:** 1 College of Veterinary Medicine, Sichuan Agricultural University, Chengdu 611130, China; 202300448@stu.sicau.edu.cn (B.W.); 202300435@stu.sicau.edu.cn (X.L.); zhouzimo@stu.sicau.edu.cn (Z.Z.);; 2 Key Laboratory of Animal Diseases and Environmental Hazards of Sichuan Province, Sichuan Agriculture University, Chengdu 611130, China

**Keywords:** mineral nutrition, female cattle, fertility, reproductive physiology, oxidative stress

## Abstract

Reproductive efficiency in female cattle is significantly influenced by micronutrient status, particularly the availability and balance of essential trace minerals. Selenium, copper, zinc, cobalt, and iron serve as critical components of enzymatic systems, antioxidant defense networks, hormone synthesis, and cellular metabolism, collectively sustaining reproductive health. This review integrates current research evidence on the physiological functions and molecular mechanisms through which these five trace minerals regulate reproductive performance in female cattle, with a specific focus on iron—an often overlooked element—highlighting the novelty of this synthesis. Both deficiency and excess of these minerals impair key reproductive outcomes such as estrous cyclicity, conception rate, and embryonic survival. Furthermore, complex interactions among minerals influence their bioavailability and physiological responses. Advances in mineral supplementation strategies, particularly the application of organic minerals and precision feeding technologies, offer promising solutions to improve reproductive performance. Elucidating these interrelationships provides a theoretical foundation for optimizing trace mineral nutrition, thereby enhancing female cattle fertility, reducing metabolic disorders and promoting the sustainable development of beef and dairy industries.

## Simple Summary

Reproduction is essential for maintaining healthy cattle herds and ensuring sustainable bovine productivity. This review explores how five key trace elements, copper, selenium, zinc, cobalt, and iron, affect the fertility of female cattle. These minerals are required only in very small amounts but have powerful effects on hormone production, pregnancy maintenance, and protection of reproductive tissues from damage. When cattle do not get enough of these elements, they may experience delayed breeding, poor conception rates, or pregnancy loss. Too much of some minerals, such as iron or copper, can also cause harm by upsetting the body’s natural balance. Understanding how these trace elements work helps farmers and veterinarians design better feeding plans, prevent reproductive problems, and improve herd productivity in an environmentally friendly way. This knowledge supports animal welfare, farm efficiency, and global food security.

## 1. Introduction

Reproductive efficiency in dairy cows is a critical determinant of profitability in the livestock industry. Low conception rates per estrous cycle, postpartum negative energy balance (NEB), retained placenta, and early embryonic mortality continue to challenge both intensive and smallholder farming systems. The multifactorial nature of reproductive inefficiency underscores the complex interactions among infectious, metabolic, nutritional, and physiological factors that regulate fertility in cattle.

Nutritional management exerts profound effects on bovine reproductive function. Nutritional management influences not only reproductive development but also the inflammatory status of the uterine environment, thereby affecting key reproductive processes such as pregnancy establishment and maintenance [1]. Research indicates that elevated dietary arginine (Arg) supplementation during bovine gestation optimizes embryo survival, thereby increasing the number of live-born calves [2]. Maternal dietary protein significantly affects fetal growth, birth weight, and related outcomes. The specific effects of maternal protein intake vary by species and stage of pregnancy, as protein requirements fluctuate during gestation to support fetal development and postpartum adaptation. For example, in beef cattle, a crude protein (CP) level of 10–12% is sufficient during early pregnancy, whereas protein levels typically increase to 12–14% in late pregnancy to support fetal growth [3]. A study by Juli Chakma et al. [4] found that supplementing cattle with an herbal formula containing leaves of Murraya koenigiiand Aegle marmeloscan enhance reproductive performance, such as advancing estrus onset and improving conception rates. A study by Castillo et al. [5] demonstrated that a mixture of condensed and hydrolyzable tannin feed additives (ByPro^®^) can improve the reproductive efficiency of lactating dairy cows. Total protein (TP) concentration serves as a reliable indicator of hepatic and renal impairment. Cattle with endometritis may exhibit elevated TP levels (e.g., 19.16 ± 1.00 g/dL), which could contribute to diminished fertility [6]. Studies have demonstrated that supplementing pregnant cattle with a combination of folic acid, methionine, and choline (OCM) enhances fetal growth, development, and metabolic processes, with potential implications for long-term offspring outcomes through fetal programming [7].

In addition to macronutrients and vitamins, trace elements play pivotal roles in maintaining reproductive health. Selenium, copper, zinc, cobalt, and iron are involved in antioxidant defense, hormone synthesis, and enzymatic reactions essential for gametogenesis and embryo development. Selenium deficiency can lead to reduced fertility, embryonic mortality, retained placenta, endometritis, and impaired overall reproductive performance in dairy cattle [6]. Copper promotes oocyte maturation and inhibits apoptosis. Zinc optimizes the reproductive hormone environment to support pregnancy maintenance. Cobalt increases progesterone and Estradiol (E2) levels, effectively shortening the postpartum anestrus period. And iron plays a key role in maintaining a normal estrous cycle and promoting reproductive hormone secretion. Meanwhile, there is also a synergistic effect between mineral elements. For instance, under heat stress (HS) conditions, selenium, copper, zinc, and iron can synergistically improve milk composition, udder health, and reproductive performance of dairy cows under heat stress [8]. Supplementing beef cattle during gestation with an organic complex of cobalt, copper and zinc can promote the growth and development of their offspring, thereby exerting long-term positive effects on the cow’s performance, health, and reproductive efficiency [9]. Balanced supplementation of these trace minerals forms an important nutritional foundation for ensuring efficient reproduction in cattle. Despite their biological importance, the reproductive roles of microminerals are often underexplored in nutritional management programs. Emerging evidence suggests that balanced mineral status can modulate ovarian follicular dynamics, corpus luteum (CL) function, and uterine receptivity, thereby influencing conception and gestation success rates. Therefore, to compile this review, a systematic and comprehensive literature search was conducted across major scientific databases and platforms. The search aimed to identify relevant, peer-reviewed studies published from January 1992 to December 2025. The search strategy utilized Boolean operators (AND, OR) to combine keywords pertaining to the five trace minerals of interest (copper, selenium, zinc, cobalt, iron), their nutritional status (deficiency or excess), and their specific effects on bovine reproductive function. This search was designed to encompass key journals in the fields of animal science, veterinary medicine, nutrition, and reproductive biology (e.g., J Anim Sci, J Dairy Sci, Anim Reprod Sci, Theriogenology, Biol Trace Elem Res, Animal, Vet Sci). The detailed search queries for each major thematic category are presented in Table 1. Although the individual roles of these minerals have been studied, a comprehensive synthesis highlighting their interactive effects, molecular mechanisms, and nutritional management strategies remains limited. By integrating findings from recent studies, this review aims to elucidate the pathways through which micromineral status modulates endocrine regulation, ovarian follicular dynamics, luteal maintenance, and embryo survival. The broader significance of this synthesis lies in its relevance to both fundamental and applied animal science. From a mechanistic perspective, it advances understanding of the biochemical and molecular interfaces between mineral metabolism and reproductive physiology. From an applied standpoint, it offers evidence-based guidance for optimizing mineral supplementation strategies to reduce infertility, shorten calving intervals, and enhance conception rates under diverse production systems. Ultimately, strengthening our understanding of micromineral interactions will contribute to more sustainable and efficient reproductive management in modern dairy herds.

## 2. The Impact of Copper on Reproductive Function in Cattle

Copper is a significant trace element involved in processes such as antioxidative defense, hormone synthesis, cell signaling, and gametogenesis, all of which are vital for the reproductive health of cattle. However, due to the unique physiological characteristics of the ruminant digestive system, the copper absorption rate in cattle is lower than that in monogastric animals. In the rumen, copper interacts with elements such as molybdenum and iron, limiting its bioavailability [10,11]—even with efficient mechanisms in cattle for regulating copper reserves, maintaining copper homeostasis, and adequate dietary copper intake, secondary copper deficiency may still occur. Copper deficiency in cattle is defined as a concentration < 30 μg/dL, and it is the second most common mineral deficiency affecting grazing cattle worldwide [12]. This condition has been demonstrated to be associated with reduced conception rates, delayed estrus, early embryonic loss, and impaired overall fertility (Table 1). Excessive copper, on the other hand, can induce oxidative stress and metabolic toxicity in reproductive tissues, further disrupting normal reproductive processes (as elaborated in Section 2.3) Therefore, an in-depth understanding of copper’s metabolic mechanisms and its regulatory effects on reproductive function, as well as the impacts of copper deficiency and excess, is of great significance for improving the fertility and production efficiency of dairy and beef cattle. According to NRC (2000) [13], the copper requirement for beef cattle is 10 mg/kg DM. NRC (2001) [14] recommends a copper requirement of 15.7 mg/kg DM for a 650 kg cow producing 40 kg of milk, while for a 700 kg non-lactating cow at 260 days of gestation, the requirement is 11.7 mg/kg DM. Thus, the copper requirement for cows at different physiological stages ranges from 10 to 15.7 mg/kg DM.

### 2.1. Role of Copper in Cumulus–Oocyte Complex (COC) Maturation

Copper deficiency impairs the antioxidative capacity of cumulus–oocyte complexes (COCs) and disrupts gap-junctional communication between cumulus cells (CCs) and oocytes, mainly due to reduced synthesis and transfer of glutathione (GSH) caused by insufficient copper-dependent enzyme activity. Copper supplementation enhances oocyte developmental competence by promoting both nuclear and cytoplasmic maturation within the COC [15]. Adequate copper availability improves gap-junctional communication between CCs and the oocyte, facilitating the transfer of GSH, a critical tripeptide responsible for maintaining intracellular redox homeostasis and preventing oxidative DNA damage. High intraoocytic GSH levels are significant for chromosomal stability and cytoplasmic maturation, while reduced oxidative stress correlates with lower apoptosis in surrounding CCs.

CC expansion, another hallmark of cytoplasmic maturation, is driven by the synthesis of hyaluronic acid-rich glycosaminoglycans incorporated into the extracellular matrix (ECM). This ECM formation within CCs is necessary for final oocyte maturation and ovulatory competence. Collectively, these findings suggest that copper contributes to oocyte maturation by modulating oxidative balance and intercellular communication within the COC, ultimately enhancing the developmental potential of bovine oocytes [16].

### 2.2. Influence of Copper on CLFunction

Copper deficiency reduces luteal antioxidant enzyme activity (e.g., glutathione peroxidase, GPx) and impairs luteal tissue growth, resulting in CL size, decreased progesterone (P4) secretion, and shortened luteal phase, which are closely linked to early embryonic loss. Copper also influences luteal development and function through its involvement in antioxidant enzyme activity and steroidogenic regulation. Luana et al. [15] reported that heifers supplemented with a copper–zinc complex exhibited higher body weight, larger CL size, and increased plasma GPx concentrations compared to controls. Notably, heifers with lower body condition scores (BCS) showed improved estrus expression and a trend toward higher pregnancy rates following copper–zinc supplementation. These findings indicate that copper, particularly in synergistic interaction with zinc, enhances luteal function and reproductive efficiency by promoting antioxidative protection and supporting luteal tissue growth.

### 2.3. Regulation of Granulosa Cell Steroidogenesis by Copper

Copper deficiency downregulates the expression of key steroidogenic enzymes and disrupts AKT/WNT signaling pathways in granulosa cells, leading to reduced E2 synthesis and impaired follicular development. Although copper does not directly promote granulosa cell proliferation, it plays a pivotal role in regulating their steroidogenic activity. Granulosa cells are the primary site of estrogen biosynthesis, driven by the coordinated expression of cytochrome P450 family enzymes and hydroxysteroid dehydrogenases (HSD). Copper activates the AKT and WNT signaling pathways, thereby upregulating the expression of cytochrome P450 family 19 subfamily A member 1 (CYP19A1) and enhancing E2 synthesis [17]. The steroidogenic acute regulatory protein (StAR) serves as a rate-limiting enzyme in this process, facilitating cholesterol transport into mitochondria for steroid hormone biosynthesis.

However, the effects of copper exhibit a concentration-dependent pattern. Lou et al. demonstrated that supplementation with 0.25 mM copper significantly increased mRNA expression of StAR, CYP11A1, 3β-HSD, and 17β-HSD, leading to elevated E2 production. In contrast, excessive copper (0.65 mM) suppressed the FSHR/CYP19A1 signaling pathway, downregulating these steroidogenic enzymes and reducing E2 synthesis [18]. Thus, maintaining optimal copper concentrations is critical to support estrogen biosynthesis while preventing oxidative or metabolic toxicity that may impair follicular function.

### 2.4. Effects of Copper on Pregnancy Rate and Fertility Outcomes

Copper deficiency increases embryonic mortality by impairing uterine receptivity and embryo implantation, as well as reducing the levels of pregnancy-associated glycoproteins (PAGs) that are essential for maintaining pregnancy. Copper’s influence extends beyond follicular and luteal physiology to overall reproductive success. Hu et al. [12] reported that copper supplementation did not significantly alter CLarea, plasma P4 concentration, or Corpus luteal blood flow (CLBF) in cattle. Nevertheless, copper-treated beef cattle showed improved pregnancy rates, while dairy cows exhibited a reduction in anovulatory P4-free follicles and plasma E2 levels. These research findings collectively suggest that copper supplementation still contributes to enhancing fertility in cattle, even in the absence of measurable changes in circulating P4.

To systematically consolidate the viewpoints, all statements from the third major section of the text are summarized in order to more intuitively and clearly illustrate the main effects and mechanisms of action of copper on the reproductive performance of cattle.

Griffiths et al. [19] demonstrated that drinking water treated with carbon glucose hexanoate and a zinc, manganese, and copper amino acid complex CTM (Critical Trace Minerals, CTM) was administered to cows to evaluate its effects on their lactation performance and fertility. The study found that CTM supplementation improved the lactation and reproductive performance of cows. This research further confirms that copper can enhance the reproductive capacity of cows.

George et al. [20] randomly assigned heifer calves to two groups. From weaning to breeding age, both groups were fed a basal diet, with one group supplemented with inorganic minerals (INORG; copper, manganese, and zinc hydroxychloride) and the other with complex minerals (COMP; copper, manganese, zinc amino acid complexes, and pentaglucose heptanoate). The results showed that neither INORG nor COMP affected estrus expression or conception rate in the cows. However, complex mineral supplementation increased hepatic cytochrome oxidase (CO) concentration, tended to elevate hepatic copper concentration, and raised the levels of PAGs, ultimately reducing embryonic mortality compared to the inorganic mineral group and improving cow fertility (Table 2).

### 2.5. Effects of Copper on the Physiological and Production Responses of Cattle and Their Offspring

Copper deficiency during late gestation impairs maternal postpartum recovery by reducing antioxidant capacity and delaying body condition restoration, and may indirectly affect offspring health by disrupting maternal nutrient metabolism. Vinicius et al. [21] randomly divided 72 non-lactating pregnant Angus cattle into three equal groups. During the third trimester of gestation, the cattle were supplemented with trace minerals (copper, manganese, and zinc) from three different sources: inorganic sulfate (INR), organic-complexed form (ORG), and hydroxychloride form (HDX). The results showed that body weight and BCS changes in cattle were similar among all groups during the supplementation period. However, cows supplemented with HDX or ORG trace minerals exhibited improved body condition at weaning, with a greater magnitude of BCS improvement from calving to weaning compared to those receiving INR trace minerals. This indicates that HDX and ORG trace minerals may exert a potential positive carry-over effect, supporting maternal postpartum recovery and body condition restoration.

Interestingly, the study revealed that the mineral concentrations in the livers of calves were not affected by the source of maternal trace mineral supplementation, suggesting that exogenous minerals did not alter the amount of minerals transmitted to the offspring. Additionally, no significant differences were observed in offspring-related outcomes, such as birth weight, growth rate, or health markers [21].

In summary, supplementation of exogenous copper (as part of combined trace minerals) to cattle during late gestation may facilitate postpartum recovery and improve maternal body condition, but it has no significant effects on the offspring’s body condition, liver mineral concentrations, or growth performance.

In conclusion, copper can enhance the developmental competence of oocytes by regulating the COC; strengthen luteal function; promote estrogen biosynthesis in granulosa cells; reduce oxidative stress; and facilitate postpartum recovery in cattle. Ultimately, it improves the pregnancy rate and reproductive capacity of cattle. Therefore, the regulation of copper on the reproductive function of cattle is of great significance.

## 3. The Impact of Selenium on Reproductive Function in Cattle

Selenium is an essential trace element that supports reproductive health in cattle primarily through its incorporation into selenoproteins. These proteins regulate key physiological processes such as hormone synthesis, antioxidant defense, and immune modulation. Selenium deficiency in cattle is mainly caused by low selenium content in forages, antagonistic interactions with sulfur, copper, and arsenic in the rumen that reduce its bioavailability, and insufficient dietary supplementation; this deficiency has been linked to impaired fertility. Meanwhile, excessive selenium intake can induce selenosis, causing oxidative damage to reproductive tissues and disrupting hormone homeostasis, which also impairs reproductive performance, whereas adequate supplementation enhances reproductive efficiency by optimizing luteal function, uterine receptivity, and oocyte quality. NRC [13] recommends selenium levels of 0.1 mg/kg DM for beef cattle and 0.3 mg/kg DM for dairy cattle, with a tolerance level of 2 mg/kg DM for selenium.

### 3.1. Selenium-Mediated Regulation of Luteal Function and Cholesterol Uptake

Selenium deficiency inhibits the low-density lipoprotein receptor (LDLR) pathway in luteal steroidogenic cells, reduces cholesterol uptake and biosynthesis-related gene expression, and decreases P4 secretion during the early luteal phase, leading to luteal insufficiency and failure of embryo implantation. Selenium modulates the expression of genes related to cholesterol metabolism in luteal steroidogenic cells, thereby promoting P4 synthesis during the early luteal phase. This process involves enhancing cholesterol uptake via the low-density lipoprotein receptor (LDLR) pathway. Studies have shown that supplementation with a balanced mixture of inorganic and organic selenium (Inorganic Selenium:Organic Selenium = 1:1) upregulates transcripts associated with cholesterol biosynthesis, leading to increased P_4_ concentrations in the early luteal phase, which is crucial for successful embryo implantation [22] (Table 3).

Wang et al. [23] investigated the effects of vitamin E (VE) and selenium on steroidogenesis in dairy cows under hydrogen peroxide-induced oxidative stress. They found that VE and selenium synergistically stimulated the proliferation of granulosa cells and enhanced the expression of steroidogenesis-related genes (StAR, HSD3β1, and CYP19A1), thereby increasing the secretion of E2 and P4. Additionally, VE and selenium reduced apoptosis and alleviated endoplasmic reticulum stress by activating the NRF2 signaling pathway, ultimately improving pregnancy rates. Soils in many major dairy regions worldwide are Se-deficient, resulting in insufficient Se content in feedstuffs; cattle fed stored forages are prone to vitamin E deficiency, a condition frequently observed in peripartum dairy cows. Deficiencies in either nutrient are associated with increased incidence and severity of intramammary infections (IMI), elevated clinical mastitis cases and somatic cell counts (SCC)—a key indicator of mastitis and milk quality. Polymorphonuclear neutrophils (PMNs) are critical for bovine mammary gland immunity, and vitamin E/Se deficiency impairs PMN activity. Supplementation with Se and vitamin E accelerates PMN influx into milk and enhances intracellular bacterial killing after intramammary bacterial challenge; subcutaneous vitamin E injections 10 and 5 days pre-calving also effectively elevate PMN alpha-tocopherol levels during the periparturient period and reverse the suppressed PMN bactericidal activity typical around calving [24].

### 3.2. Effects of Selenium on the Bovine Endometrium

Selenium deficiency weakens the antioxidant defense system of endometrial cells, reduces the activity of the Nrf2 signaling pathway, increases reactive oxygen species (ROS) accumulation, inhibits the expression of TGF-β1 and TGF-β3, blocks the AKT/GSK-3β and Wnt/β-catenin signaling pathways, hinders endometrial repair, and elevates the risk of postpartum metritis and embryo implantation failure. Lipopolysaccharide (LPS) inhibits cell viability, induces apoptosis, downregulates the mRNA expression of connective tissue growth factor (CTGF), thereby transforming growth factor-β1 (TGF-β1) and transforming growth factor-β3 (TGF-β3), and suppresses the AKT and WNT signaling pathways, thereby impairing endometrial repair. Elevated cortisol levels further exacerbate LPS-induced impairments in cell proliferation and apoptosis.

Dong et al. [25] conducted combined treatment experiments with LPS, cortisol, and selenium on bovine endometrial stromal cells (BESCs). The results showed that selenium supplementation promotes cell proliferation, attenuates apoptosis, upregulates the mRNA expression of TGF-β1 and TGF-β3, and activates the AKT/GSK-3β and Wnt/β-catenin signaling pathways—ultimately facilitating the growth of LPS-injured BESCs under high cortisol conditions.

Cui et al. [26] pretreated primary BESCs with selenium for 12 h and found that selenium upregulates key molecules of the Nrf2 pathway, significantly increasing the mRNA and protein expression levels of NFE2L2 (encoding the Nrf2 protein), HMOX1, and NQO1 in BESCs (*p* < 0.05). This enables BESCs to resist oxidative stress induced by factors such as LPS and high cortisol. Meanwhile, selenium significantly enhances the activities of superoxide dismutase (SOD), GPx, and catalase (CAT) in LPS-stimulated BESCs, increases GSH content (*p* < 0.05), and reduces the accumulation of ROS and malondialdehyde (MDA) (*p* < 0.01), ultimately alleviating oxidative stress in primary BESCs. In addition, selenium promotes the migration of Nrf2 from the cytoplasm to the nucleus, which helps initiate the transcription of downstream antioxidant genes and mitigate oxidative stress-induced damage to the endometrium.

In summary, these findings indicate that selenium supplementation facilitates the rapid repair of the endometrium and alleviates damage in postpartum dairy cows.

### 3.3. Effects of Selenium on Interferon

Selenium deficiency leads to excessive activation of the JAK/STAT1/2 signaling pathway in the endometrium, causing abnormal overexpression of interferon-stimulated genes (ISGs), triggering excessive maternal immune responses, inducing maternal recognition of the fetus as a “foreign body”, and increasing the risk of early embryonic loss. Interferon-τ (IFN-τ) secreted by bovine embryonic trophoblast cells can bind to endometrial receptors, inhibit the transcription of estrogen receptors (ER), block the release of luteolytic factors, and maintain P4 secretion, thereby improving the successful implantation of embryos [27]. Additionally, it activates the JAK/STAT1/2 signaling pathway and induces the expression of ISGs [28]. When IFN-τ and ISGs are used as diagnostic and prognostic biomarkers for maternal–fetal cell crosstalk, ISGs have been proven to be the optimal peripheral biomarkers for predicting pregnancy outcomes and embryonic mortality during the peri-implantation period [29]. However, excessive ISG activation can trigger maternal immune hyperactivation, leading the mother to recognize the fetus as a “foreign body” and initiate a rejection response [28]. Selenium can inhibit excessive ISG expression, prevent the maternal immune system from rejecting the fetus, and simultaneously ensure the normal signal transduction of IFN-τ, thereby supporting embryonic implantation and early pregnancy maintenance.

**Table 3:** Selenium: Effects and mechanisms on reproductive function in cattle.

Resource	Animal/Cell	Major Effects	Mechanism	Reference
Inorganic selenium (sodium selenite, sodium selenate), organic selenium (selenomethionine, selenocysteine) 1:1	Angus crossbred Dairy Cows Luteal steroidogenic cells	1:1 mixture supplementation promotes early luteal-phase P4 synthesis, enhances embryo implantation, upregulates cholesterol biosynthesis transcripts	Modulates luteal cell cholesterol metabolism, enhances LDLR-mediated uptake	[23]
Selenium + VE	Dairy Cows	Under H_2_O_2_-induced oxidative stress: synergistically stimulates granulosa cell proliferation, enhances steroidogenesis-related gene expression (StAR, HSD3β1, CYP19A1), increases E2/P4 secretion, reduces granulosa cell apoptosis and endoplasmic reticulum stress, improves pregnancy rate	Regulates steroid hormone synthesis pathway	[24]
Sodium selenite (Na_2_SeO_3_)	Holstein BESCs	Resists LPS/high cortisol-induced oxidative stress (12 h pretreatment); increases SOD/GPX/CAT activity, GSH content; reduces ROS/MDA; promotes cell proliferation; attenuates apoptosis; facilitates the growth of LPS-injured BESCs	Activates Nrf2 pathway, upregulates NFE2L2/HMOX1/NQO1, promotes Nrf2 nuclear translocation	[26]
Selenium (form not specified)	BGCs	Reduces apoptosis, oxidative and endoplasmic reticulum stress; enhances T-AOC; promotes proliferation; facilitates oocyte maturation	Activates Nrf2 pathway, upregulates NQO1/HO-1/GCLM/GCLC, inhibits ROS/MDA production	[25,30]
Inorganic–organic selenium mixture (1:1)	Angus Cattle	Supplementation with 1:1 mixture downregulates JAK/STAT1/2 pathway, reduces ISGs (IFIT1, IFIT2, IRF9) expression, avoids maternal immune rejection, preserves IFN-τ core function	Inhibits STAT2/IRF9 expression, blocks STAT1-STAT2-IRF9 complex formation	[27,28]

Sarah et al. [28] conducted a mixed selenium (MIX) supplementation experiment on grazing beef cattle, using a 1:1 combination of inorganic and organic selenium. The results showed that MIX significantly downregulated the activity of the JAK/STAT1/2 pathway (*p* < 0.05) and reduced the mRNA expression of downstream classical ISGs, including IFIT1, IFIT2, and IRF9. Specifically, the mechanism involves selenium inhibiting the expression of STAT2 and IRF9, thereby blocking the formation of the “STAT1-STAT2-IRF9” transcriptional complex. This prevents the complex from binding to the promoters of ISGs, ultimately reducing the transcriptional expression of ISGs. This regulatory effect not only preserves the core function of IFN-τ but also avoids excessive immune activation, creating favorable conditions for the embryo to escape maternal immune rejection.

### 3.4. Effects of Selenium on Granulosa Cells and Oocytes

Selenium deficiency reduces the antioxidant capacity of granulosa cells and oocytes, inhibits the Nrf2 signaling pathway, increases the accumulation of ROS and MDA, promotes granulosa cell apoptosis, blocks the maturation of COCs, reduces the fertilization rate and embryonic developmental potential. Granulosa cells play protective, regulatory, and nutritive roles in oocyte maturation. They mainly provide nutritional support for oocytes, and more importantly, bovine granulosa cells (BGCs) regulate oocyte development by secreting steroid hormones. Selenium can reduce oxidative stress in granulosa cells, thereby better ensuring the development and nutritional supply of oocytes [18].

Wang et al. [25] found that selenium downregulates the expression of apoptosis-related genes, thereby reducing granulosa cell apoptosis. Meanwhile, selenium inhibits the production of ROS and MDA, enhances total antioxidant capacity (T-AOC), and improves the activities of SOD, CAT, and GPx, thus alleviating oxidative stress. Furthermore, selenium reduces endoplasmic reticulum stress by activating nuclear factor erythroid 2-related factor 2 (Nrf2) and upregulating the expression of its downstream genes (NQO1, HO-1, GCLM, and GCLC). This experiment demonstrated that selenium promotes the proliferation of bovine granulosa cells and reduces granulosa cell apoptosis and oxidative stress by activating the Nrf2 signaling pathway, thereby facilitating oocyte maturation [25].

Additionally, Shervin et al. [30] evaluated the potential protective effects of selenium supplementation on the maturation and subsequent development of bovine COCs exposed to HS by adding sodium selenite (Na_2_SeO_4_) to the in vitro maturation (IVM) medium under heat stress conditions. The study found that selenium can activate the NRF2 signaling pathway to regulate the expression of antioxidant genes; as a core component of the GPx family of antioxidant enzymes, it participates in cellular defense against oxidative stress; downregulates the expression of oxidative stress-related enzymes and markers (SOD, CAT, and GPX-4) in CCs and oocytes; and reduces endoplasmic reticulum stress. The results demonstrated that selenium improved the maturation rate and subsequent developmental competence of bovine oocytes under HS conditions, and alleviated the adverse effects of HS on the viability of bovine oocytes and granulosa cells. Meanwhile, selenium enhanced the viability of CCs, upregulated the expression of antioxidant candidate genes, and downregulated the expression of apoptosis-related genes.

Selenium plays a central regulatory role in bovine reproductive physiology. Therefore, when assessing the impact of its deficiency on specific production-related disorders such as retained placenta, the production type of cattle must be fully considered. It is important to note that the effect of selenium deficiency on retained placenta is particularly pronounced in dairy cattle, especially high-yielding lactating cows, whereas it is of relatively minor significance in beef cattle. This distinction stems primarily from fundamental differences in their periparturient metabolic load. Dairy cows experience extreme negative energy balance and oxidative stress around calving, making their reproductive tissues critically dependent on selenium-dependent antioxidant and anti-inflammatory protective mechanisms. Consequently, insufficient selenium supply compromises uterine repair and normal fetal membrane detachment in dairy cows, leading to a significantly elevated incidence of retained placenta. In contrast, beef cattle experience lower metabolic stress. According to the NRC [31], Nutrient Requirements of Beef Cattle, and established clinical practice, selenium deficiency in this group more commonly presents with other symptoms, such as white muscle disease, with retained placenta not being a typical or sensitive clinical indicator.

Therefore, in establishing selenium nutritional requirements, diagnostic criteria for deficiency, and supplementation strategies, it is essential to strictly differentiate between the distinct physiological states, metabolic pressures, and management objectives of dairy and beef cattle to implement precise nutritional management.

In summary, selenium can regulate bovine reproductive function through multiple pathways: improving luteal function; promoting estrogen release; inhibiting excessive expression of ISGs to prevent maternal immune rejection of the fetus; reducing oxidative stress and heat stress in granulosa cells; and facilitating oocyte development, among other effects. Therefore, selenium plays a crucial regulatory role in the reproductive function of female cattle.

## 4. The Impact of Zinc on Reproductive Function in Cattle

Zinc is the third most commonly deficient trace element in grazing cattle and participates in various fundamental biological processes, including hormone secretion, antioxidant defense, and immune regulation (Table 4). Zinc deficiency in cattle is mainly caused by low zinc content in forages, formation of insoluble complexes with calcium, phosphorus, and phytate in the rumen that reduce its bioavailability, and increased metabolic demand in high-yielding dairy cows; in extreme cases, zinc deficiency may result in abortion or the birth of weak calves [32]. Excessive zinc intake, however, can interfere with the absorption and utilization of copper and iron in the rumen, induce oxidative damage to ovarian and uterine tissues, and disrupt hormone homeostasis, thereby impairing reproductive performance. A plasma zinc concentration above 90 μg/dL is considered adequate, 80–90 μg/dL indicates marginal deficiency, and below 80 μg/dL signifies zinc deficiency [33]. Among diagnostic indicators, alkaline phosphatase activity is considered a more sensitive biomarker [33]. NRC [13] recommends a zinc level of 30 mg/kg dry matter (DM) for both pregnant beef cows and finishing cattle, with a maximum tolerance concentration of 500 mg/kg DM.

### 4.1. Regulation of Hormone Secretion and Function by Zinc

Zinc deficiency destabilizes the structure of zinc-finger proteins (nuclear receptors for steroid hormones), reduces the activity of steroid hormone receptors and gonadotropin receptor sensitivity, disrupts balanced hormone secretion and CL function, and further leads to reproductive disorders in Aberdeen Angus cows, including abnormal ovarian development, irregular estrous cycles, and delayed follicular growth. Zinc acts as a cofactor in numerous enzymes, functions as a component of a wide range of transcription factors, and is involved in nearly every signaling pathway in the mammalian body [33,34,35,36]. Zinc optimizes the hormonal environment mainly by serving as an essential component of zinc-finger proteins (nuclear receptors for steroid hormones) to maintain receptor stability and activation, while also enhancing gonadotropin receptor sensitivity for balanced hormone secretion and CLfunction [37]. Zinc deficiency—whether mild or severe—can lead to reproductive disorders in Aberdeen Angus cows, including abnormal ovarian development, irregular estrous cycles, and delayed follicular growth [38].

Anchordoquy et al. [38] demonstrated that zinc supplementation increases CLarea in deficient Aberdeen Angus cows and can even raise plasma P4 levels in normal cows. Adequate P4 is critical for early pregnancy, as its concentration correlates positively with placental growth during this period [39]. Early P4 supplementation promotes embryonic development and improves uterine secretory function; levels below 2.8 ng/mL may reduce conception rates by up to 50% [39].

During the periparturient period, zinc sulphate heptahydrate combined with vitamin E helps stabilize circulating hormones and biochemical markers such as insulin, prostaglandins, and IGF-1 in peri-partum Sahiwal cows, thereby alleviating parturition-related stress [40]. Messersmith et al. [17] further noted that zinc modulates hormone receptor signaling and affects the secretion and bioavailability of IGF-1, which influences uterine involution, embryo implantation, and fetal growth [17].

### 4.2. Antioxidant Defense Role of Zinc

Zinc deficiency impairs the activity of copper/zinc superoxide dismutase, inhibits the SIRT1/PGC-1α pathway, aggravates mitochondrial dysfunction, increases intracellular ROS accumulation, induces oxidative damage to oocytes and luteal cells, and reduces blastocyst quality and developmental potential. Zinc mitigates mitochondrial dysfunction by mediating mitochondrial biogenesis and upregulating the expression of the SIRT1/PGC-1α pathway, thereby reducing oxidative stress and apoptosis [41]. It can also attenuate arsenic-induced intracellular oxidative stress by upregulating metallothionein [42], and zinc oxide nanoparticles enhance the synthesis of the antioxidant GSH by increasing GCLM gene expression in bovine intestinal epithelial cells (BIECs) [43]. Furthermore, zinc is an integral structural component of copper/zinc superoxide dismutase, a key enzyme that alleviates oxidative stress and maintains the structural integrity of the CL [44,45,46,47]. By scavenging ROS, copper/zinc superoxide dismutase prevents cellular damage and promotes P4 synthesis [48]. The binding of zinc ions to its active site is crucial for its full enzymatic activity. An in vitro study by Yun Feng et al. [49] revealed that Supplementation with 0.8 μg/mL zinc sulfate enhanced antioxidant defense by activating the Nrf2 pathway, stabilized mitochondrial function and DNA integrity, and ultimately improved blastocyst quality and developmental outcomes in Limousin cattle bovine oocytes.

### 4.3. Immune Enhancement by Zinc

Zinc deficiency reduces the phagocytic activity of peripheral blood leukocytes, upregulates the expression of pro-inflammatory cytokines, weakens the anti-inflammatory defense mechanism of the reproductive tract, increases the risk of postpartum metritis and abortion, and hinders reproductive recovery. Zinc supplementation also enhances immune function, reducing reproductive tract inflammation and the risk of abortion. Dang et al. [50] reported that zinc sulfate increased the phagocytic activity of peripheral blood leukocytes in postpartum cows, thereby improving immune competence and aiding reproductive recovery. Mohammadi et al. [51] found that zinc supplementation (Zinc Gluconate, Zinc Sulfate) could regulate the expression levels of C-reactive protein and interleukin-6, exerting anti-inflammatory effects in vivo. Moreover, zinc oxide may enhance the host’s non-specific defense mechanisms through immune modulation, increasing the percentages of lymphocytes and monocytes while decreasing the neutrophil percentage [42]. Nano-zinc oxide can promote the expression of antioxidant-related genes (HO-1 and GCLM) and the anti-inflammatory cytokine gene IL-10, while suppressing the expression of pro-inflammatory cytokine genes in Bovine Intestinal Epithelial Cells [43].

**Table 4:** Zinc: Effects and mechanisms on reproductive function in cattle.

Resource	Animal/Cell	Major Effects	Mechanism	Reference
Zinc sulphate heptahydrate (ZnSO_4_·7H_2_O) + VE	Periparturient Cows	1. Stabilizes periparturient hormones, alleviates parturition-related stress 2. Enhances reproductive performance, promotes uterine involution, embryo implantation and fetal growth	1. Regulates gonadotropin secretion and hormone receptor signaling (IGF-1) 2. Maintains reproductive tract mucosal integrity, boosts antioxidant capacity	[17,40]
Zinc oxide nanoparticles (nano-ZnO)	BIECs	1. Enhances antioxidant GSH synthesis 2. Upregulates antioxidant (HO-1, GCLM) and anti-inflammatory (IL-10) genes 3. Downregulates pro-inflammatory cytokine genes (IL-6, IL-8)	1. Increases GCLM gene expression (for GSH synthesis) 2. Regulates expression of antioxidant and inflammatory cytokine genes	[43]
Zinc sulfate (ZnSO4)	Limousin Cattle Bovine Oocytes; Bovine Blastocysts (in vitro)	0.8 μg/mL in vitro supplementation: Enhances antioxidant defense; stabilizes mitochondrial function and DNA integrity; improves blastocyst quality and developmental outcomes (0.8 μg/mL)	Activates Nrf2 pathway	[49]
	Postpartum Cows	Increases phagocytic activity of peripheral blood leukocytes, enhances postpartum immune competence, aids reproductive recovery	Enhances peripheral blood leukocyte phagocytosis, modulates inflammatory cytokine expression	[50]
Zinc–manganese AAC	Periparturient dairy cows	1. Higher bioavailability than INO forms 2. Alleviates oxidative stress/infection risks, reduces postpartum reproductive diseases (retained placenta, metritis) 3. Mitigates oocyte/CL oxidative damage, supports postpartum estrus/ovulation/conception	1. Enhances total antioxidant capacity and PMN phagocytic activity 2. Improves liver function/energy metabolism (reduced γ-glutamyl transferase, decreased ketone bodies, increased DMI) 3. Ensures normal reproductive hormone synthesis/metabolism	[52]
Zinc (in vitro)	Bovine embryos (in vitro)	Direct zinc exposure reduces blastocyst formation rate (impairs implantation potential); successfully implanted embryos show increased birth weight (altered developmental synchrony)	Disrupts embryonic development-uterine microenvironment synchrony	[53]
1,10-phenanthroline (PHEN, zinc chelator)	Bovine oocytes (in vitro activation)	0.5 mM PHEN for 1 h (zinc chelation alone): Induces blastocyst formation with compromised quality and aberrant cell lineage specification; calcium signaling indispensable for bovine embryonic competence	Reveals species divergence in zinc flux requirement (bovine oocyte activation dependent on calcium, unlike swine)	[54]
Curcumin-functionalized zinc oxide nanoparticles (ZnO(np) + CUR)	Bovine oocytes; bovine blastocysts (IVM)	6 µM/12 µM in vitro supplementation: Significantly enhances blastocyst production rate; improvement not linearly correlated with canonical antioxidant markers (ROS/SOD)	Unclear (blastocyst rate improvement decoupled from classic oxidative stress indices)	[17]
Copper–zinc (CuZn) solution (15 mg/mL Cu, 50 mg/mL Zn)	Nellore heifers (fixed-time AI, FTAI)	5 mL per heifer subcutaneous injection (9 days pre-FTAI): Improves body weight and estrus expression scores; tends to increase pregnancy rates (predominantly in low BCS heifers, BCS < 5)	Modulates reproductive performance in a body condition-dependent manner	[17]

During the periparturient period in dairy cows, supplementing zinc and manganese as amino acid complexes (AAC) shows higher bioavailability than inorganic sulfate (INO) forms. The core mechanism is synergistically regulating body homeostasis via multiple pathways: enhancing total antioxidant capacity and polymorphonuclear neutrophil phagocytic activity, improving liver function and energy metabolism (characterized by reduced γ-glutamyl transferase levels, decreased ketone body accumulation and increased dry matter intake). This effectively alleviates oxidative stress and infection risks, reduces the incidence of postpartum reproductive diseases such as retained placenta and metritis, mitigates oxidative damage to oocyte and corpus luteum function, and ensures normal synthesis and metabolism of reproductive hormones, thus laying a favorable physiological foundation for postpartum resumption of estrus, ovulation and conception [52].

### 4.4. The Effects of Zinc on Embryonic Implantation

The role of zinc in mammalian reproduction is complex and stage-specific, with its effects critically dependent on concentration, chemical form, and species. During IVM, supplementation with curcumin-functionalized zinc oxide nanoparticles (ZnO(np) + CUR) at concentrations of 6 µM and 12 µM significantly enhances the blastocyst production rate from bovine oocytes, an improvement not linearly correlated with canonical antioxidant markers like reduced ROS or increased SOD activity [53]. Following maturation, the requirement for zinc flux during oocyte activation demonstrates remarkable species divergence. In swine, zinc chelation alone using 1,10-phenanthroline (PHEN) at 1 mM for 30 min is sufficient for effective activation, yielding high-quality blastocysts. Conversely, bovine oocytes activated solely via zinc chelation (e.g., 0.5 mM PHEN for 1 h) develop into blastocysts with compromised quality and aberrant cell lineage specification, underscoring the indispensable role of calcium signaling for bovine embryonic competence [54]. Post-activation, the impact of zinc remains nuanced. Direct exposure of bovine embryos to zinc during in vitro culture is reported to reduce the blastocyst formation rate, potentially impairing implantation competence; however, embryos that successfully implant can develop to term with increased birth weights, suggesting altered developmental synchrony [17]. In vivo, systemic zinc status profoundly influences reproductive outcomes. A single subcutaneous injection of a copper–zinc (CuZn) solution (containing 15 mg/mL copper and 50 mg/mL zinc, at 5 mL per heifer) administered 9 days before fixed-time artificial insemination (FTAI) to Nellore heifers improved body weight, estrus expression scores, and tended to increase pregnancy rates, particularly in animals with a low body condition score (BCS < 5) [17]. Collectively, these findings highlight zinc as a pivotal, multi-faceted regulator across the female reproductive continuum, whose precise effects must be carefully contextualized within the specific developmental window, species physiology, and metabolic status.

## 5. Effects of Cobalt on Reproductive Function in Cattle

As ruminants, cattle rely on rumen microorganisms to synthesize vitamin B12 (cobalamin) using cobalt, which also supports rumen bacterial growth and contributes to the composition of adenosylcobalamin and methylcobalamin [55]. Cobalt deficiency in cattle is mainly caused by low cobalt content in forages grown on cobalt-deficient soils, excessive intake of antagonists such as iron and manganese that compete with cobalt for rumen microbial absorption, and insufficient dietary supplementation; this deficiency can lead to impaired vitamin B12 synthesis, disrupted rumen fermentation, and further reproductive dysfunction. Excessive cobalt intake can accumulate in the liver and kidneys, induce oxidative damage to reproductive tissues such as the ovaries and uterus, disrupt steroid hormone synthesis, and increase the risk of embryonic loss and obstetric diseases. As an essential trace element, cobalt supplementation at appropriate levels significantly improves reproductive performance in cows, primarily by optimizing hormonal balance and enhancing metabolic function [56]. Cobalt deficiency is a common mineral deficiency in herbivores [57]. Therefore, cobalt plays an irreplaceable role in the reproductive system function of cattle (Table 5). Through vitamin B12-dependent pathways, cobalt regulates the hypothalamic–pituitary–ovarian (HPO) axis, directly influencing the synthesis and secretion of reproductive hormones [58], which is the core mechanism by which it improves reproductive performance. NRC [13] recommends a cobalt requirement of approximately 0.10 mg/kg DM for beef cattle, with a dietary concentration of 0.15–0.25 mg/kg DM suggested in beef cattle rations.

### 5.1. Effects of Cobalt on Reproductive Metabolism-Associated Hormones

Cobalt deficiency reduces the activity of rumen microorganisms responsible for vitamin B12 synthesis, impairs the function of the HPO axis, decreases the secretion of P4 and E2, prolongs the postpartum estrus interval, and increases the risk of early embryonic loss. Adding 0.32 mg/kg cobalt to the diet of Magrabi she-camels (from 3 months before calving to 9 months after calving) resulted in significantly elevated serum P4 and E2 levels (compared to the non-supplemented group). The increased P4 levels reduced the risk of early embryonic loss, while E2 shortened the postpartum estrus interval by promoting follicular development and endometrial hyperplasia. Concurrently, serum thyroid hormone (T3, T4) levels in the cobalt-supplemented group also increased, providing energy support for reproductive system metabolism and indirectly optimizing estrous cycle rhythms [59]. Previous studies have stratified and randomly assigned 24 Holstein dairy cows (10 primiparous and 14 multiparous, with a lactation period of 238 ± 68.8 days) into groups based on milk production, the control group was fed a total mixed ration containing 12.5 mg of cobalt per cow per day (cobalt carbonate), while the experimental group (Co-LAC) was additionally supplemented with 50 mg of cobalt per cow per day as cobalt lactate. After a 7-day covariate period, data were collected for 4 weeks. The results showed no significant differences in milk production (26.2 vs. 25.8 kg/d), dry matter (DM) intake (22.9 vs. 23.1 kg/d), milk component yields, and body weight (684 vs. 674 kg) between the two groups. However, the rumen ammonia concentration in the Co-LAC group was lower (12.3 vs. 15.8 mg/dL), and the molar concentration of acetic acid was higher (61.1% vs. 59.5%), indicating that the additional supplementation of cobalt lactate did not improve lactation performance but could regulate rumen fermentation [60].

In addition, after participating in vitamin B12 synthesis, cobalt enhances the activity of steroid hormone synthases (such as CYP11A1, CYP19A1, and 3β-HSD), thereby promoting the conversion of cholesterol into P4 and E2 [18]. However, its effects are dual-sided. Lou et al. demonstrated that 0.02 mM cobalt significantly induced granulosa cell synthesis of E2, 0.15 mM cobalt significantly inhibited E2 synthesis, and both 0.08 mM and 0.15 mM cobalt significantly suppressed P4 synthesis. Regarding gene expression effects, 0.02 mM cobalt significantly upregulated StAR, CYP11A1, and 17β-HSD gene expression while significantly downregulating CYP19A1 expression; 0.15 mM cobalt significantly downregulated StAR, CYP11A1, 3β-HSD, and 17β-HSD gene expression [18]. These genes encode enzymes involved in estrogen and androgen synthesis, thereby influencing sex hormone balance [61,62,63].

### 5.2. Effects of Cobalt on Nitrogen Metabolism in Hormone Synthesis

Cobalt deficiency disrupts rumen microbial nitrogen metabolism, reduces the availability of amino acids required for protein synthesis, and further impairs the material basis for reproductive hormone synthesis, leading to imbalanced hormone secretion and reduced reproductive performance. In the organic cobalt replacement trial, the treatment group (partial replacement of inorganic zinc, copper, manganese, and cobalt with organic forms, with 100% cobalt replacement) did not directly alter mineral element concentrations in the CL (*p* > 0.1). However, it significantly increased milk urea nitrogen content (*p* = 0.039), indirectly reflecting optimized nitrogen metabolism. Nitrogen metabolism is closely linked to protein synthesis, providing the material basis for hormone synthesis. Furthermore, in this trial, the treatment group exhibited significantly increased milk yield at 14 weeks postpartum (*p* = 0.047) and a trend toward increased milk yield at 13 weeks (*p* = 0.089). The improvement in milk quality indicators formed a positive feedback loop with reproductive hormone balance, confirming that cobalt achieves synergistic optimization of reproductive and lactation functions through hormonal regulation [64]. Although moderate cobalt supplementation is beneficial, abnormally elevated cobalt levels (such as those observed in environmental pollution or pathological conditions) may be associated with reproductive disorders in cows. Uterine torsion is a severe obstetric emergency associated with high mortality rates [65]. In one study, serum cobalt concentrations in newborn calves of cows experiencing uterine torsion were significantly higher than those in calves of unaffected cows (*p* = 0.01) [66].

Interestingly, cobalt disrupts iron metabolism, which is critical for male germ cell differentiation, so we investigated the effects of developmental chronic cobalt exposure on mouse testes via altered Fe homeostasis in adulthood. Pregnant ICR mice were exposed to cobalt chloride (CoCl_2_) in drinking water at 75 mg/kg (low dose) or 125 mg/kg (high dose) of body weight 3 days before delivery, with treatment continuing until the offspring reached postnatal day 90, while age-matched controls received regular tap water. Testicular analyses were performed using immunohistochemistry, inductively coupled plasma mass spectrometry, and sperm counting, which showed that chronic CoCl_2_ administration caused dose-dependent cobalt accumulation in the serum and testes of exposed mice, accompanied by increased Fe levels (notably, testicular iron content in low-dose mice was ~2.7-fold higher than that in high-dose mice), dose-dependent reductions in relative testicular weight by 18.8% (low dose) and 37.7% (high dose), germ cell loss, reduced sperm count, disrupted Sertoli cell androgen responsiveness, altered localization and expression of iron metabolism-related proteins (ferroportin, hepcidin, transferrin receptor 1 [TfR1], divalent metal transporter 1 [DMT1]), and identification of Leydig cells as key sites for testicular iron metabolism. These findings led to the conclusion that cobalt exerts indirect detrimental effects on testes by disrupting iron homeostasis. Currently, there are no relevant studies on cattle [67,68,69].

**Table 5:** Cobalt: Effects and mechanisms on reproductive function in cattle.

Resource	Animal/Cell	Major Effects	Mechanism	Reference
Cobalt	Magrabi she-camels	1. Supplementation at 0.32 mg/kg increases serum progesterone E2, T3, and T4 levels 2. Reduces early embryonic loss risk and shortens postpartum estrus interval	1. Regulates HPO axis via vitamin B12-dependent pathways 2. Enhances steroid hormone synthase activity CYP11A1, CYP19A1, and 3β-HSD	[18,59]
Cobalt (as Chloride, Cobaltous)	Bovine Granulosa Cells (in vitro)	Concentration-dependent effects: 0.02 mM induces E2 synthesis 0.08 & 0.15 mM suppress P4 synthesis 0.15 mM inhibits E2 synthesis	Modulates expression of steroidogenic genes (StAR, CYP11A1, 3β-HSD, 17β-HSD, CYP19A1).	[18]
Cobalt carbonate (CoCO_3_)	Holstein Dairy Cows	12.5 mg/head/day basal dietary supplementation shows no significant differences in milk production, DMI, milk components, or body weight compared to cobalt lactate-supplemented group	Provides basal cobalt nutrition for rumen function and metabolism	[60]
Cobalt lactate [Co(C_3_H_5_O_3_)_2_]	Holstein Dairy Cows	50 mg/head/day additional dietary supplementation reduces rumen ammonia concentration, increases molar concentration of acetic acid, regulates rumen fermentation (no improvement in lactation performance)	Modulates rumen microbial fermentation pathways	[60]
Organic cobalt	Cows	1. 100% organic cobalt replacement (partial inorganic Zn/Cu/Mn replacement) increases postpartum 14-week milk yield and milk urea nitrogen content; optimizes nitrogen metabolism 2. Synergistically improves reproductive and lactation functions via hormonal balance	1. Provides material basis for hormone synthesis and nitrogen metabolism optimization 2. Regulates reproductive hormone balance, positive feedback with lactation	[64]
CoCl_2_	ICR mice (offspring, developmental chronic exposure)	75 mg/kg BW (low dose) & 125 mg/kg BW (high dose) in drinking water (3 days pre-delivery to postnatal day 90): 1. Dose-dependent Co accumulation in serum and testes, accompanied by increased testicular Fe levels (low-dose Fe content ~2.7-fold higher than high-dose) 2. Dose-dependent reduction in relative testicular weight (18.8% for low dose, 37.7% for high dose) 3. Germ cell loss, reduced sperm count, disrupted Sertoli cell androgen responsiveness 4. Altered localization and expression of Fe metabolism-related proteins (ferroportin, hepcidin, TfR1, DMT1)	1. Disrupts Fe homeostasis in testes 2. Indirectly exerts detrimental effects on testicular function via Fe metabolism disorder 3. Leydig cells are identified as key sites for testicular Fe metabolism disruption	[67,68,69]

## 6. Effects of Iron on Reproductive Function in Cattle

As an essential trace element in cattle, iron profoundly influences the physiological regulation of the reproductive system by modulating key processes such as hormone synthesis, oxidative stress balance, and placental substance transport (Table 6). Iron deficiency in cattle is mainly caused by low iron content in forages, formation of insoluble complexes with phytate, calcium, and phosphorus in the rumen that reduce its bioavailability, increased iron demand during late gestation and lactation, and parasitic infections that induce chronic blood loss; this deficiency disrupts steroid hormone synthesis and oxidative stress balance, further impairing reproductive function. Excessive iron intake can induce iron overload in the body, compete with copper for absorption sites in the intestinal tract, reduce copper bioavailability, and trigger ferroptosis in reproductive tissues such as the ovaries and uterus, thereby increasing the risk of follicular atresia and early embryonic loss. NRC [13] recommends an iron requirement of approximately 50 mg/kg dry matter (DM) for beef cattle. For normal growth, calves require 120 mg/kg DM of iron. As the animals develop, the dietary iron requirement can be gradually reduced to 50 mg/kg DM.

### 6.1. Positive Effects of Iron on the Bovine Reproductive System

Iron deficiency reduces the activity of cytochrome P450 family steroid hormone synthesis enzymes, decreases the secretion of luteinizing hormone (LH) and E2, disrupts the regularity of estrous cycles, and increases the incidence of recurrent estrus in cows. Iron also interacts synergistically with copper in regulating ovarian function: both are involved in the synthesis and activation of antioxidant enzymes and steroid hormone synthases, and their balanced ratio is crucial for maintaining normal follicular development and CL formation. As a component of steroid hormone synthesis enzymes (e.g., cytochrome P450), iron promotes the secretion of LH and E2, thereby maintaining regular estrous cycles [70]. Research involving 10 Holstein-Friesian crossbred cows with normal estrus cycles and 10 with recurrent estrus showed that serum iron and phosphorus levels in the recurrent estrus group were significantly lower than in the normal group (*p* < 0.05) when measured spectrophotometrically 12 h post-estrus. TP levels showed no intergroup differences, indicating a correlation between recurrent estrus and iron/phosphorus levels [71]. In Holstein dairy cows, iron deficiency has been found to correlate significantly with delayed luteal activity. Specifically, the study demonstrated that by monitoring postpartum cows (experiment initiated at 2 weeks postpartum, including 100 Holstein cows with 2–5 parity, average BCS 3.0 ± 0.25), serum iron concentrations were significantly reduced from 22.74 ± 0.82 (µmol/L) to 18.33 ± 1.01 (µmol/L) (*p* < 0.05) in individuals with delayed luteal activity. This suggests iron deficiency may impair ovarian function, affecting CL formation and maintenance, thereby indirectly contributing to reduced fertility [72].

### 6.2. Effects of Iron Excess on Bovine Reproductive System

#### 6.2.1. Effects of Iron on Ovarian Structural and Functional Impairment

Iron overload induces morphological alterations in the ovaries. The following conclusions are based on mouse models and have not yet been validated in cattle. In mouse studies, excessive iron intake (0.5 g/kg and 1.0 g/kg weekly for 8 weeks) resulted in significantly reduced ovarian volume accompanied by impaired follicular development. Histological examination revealed increased numbers of atretic follicles, reduced CL, and ovarian fibrosis positively correlated with serum iron levels [71]. At present, there are no relevant studies about cattle.

**Table 6:** Iron: Effects and mechanisms on reproductive function in cattle.

Resource	Animal/Cell	Upper Limit Value	Major Effects	Mechanism	Reference
Iron	Holstein-Friesian crossbred cows	\	1. Promotes LH E2 secretion, maintains regular estrous cycles 2. Recurrent estrus group has lower serum iron levels than normal group	Acts as component of steroid hormone synthesis enzymes such as cytochrome P450	[70,71]
	Holstein dairy cows		1. Iron deficiency correlates with delayed luteal activity and reduced fertility 2. Delayed luteal activity individuals have significantly reduced serum iron concentrations, reduced from 22.74 ± 0.82 µmol/L to 18.33 ± 1.01 µmol/L (*p* < 0.05)	Iron deficiency impairs ovarian function, affects CL formation and maintenance	[72]
	Bovine Theca cells		Excess iron inhibits theca cell ferroptosis via GPX4 pathway leads to diminished ovarian reserve	Modulates ferroptosis pathway related to premature ovarian failure	[73]
Ferrous ion	Bovine Granulosa cells		Ferrous ion inhibits granulosa cell proliferation and arrests cell cycle	Modulates ROS-mediated p38MAPK, p53 and p21 pathways (mechanism not investigated in cows)	[73]
Iron dextran	Mouse Ovaries (in vivo)	0.5 g/kg & 1.0 g/kg weekly (8 weeks, iron overload/toxic dose)	Iron overload reduces ovarian volume, impairs follicular development, increases atretic follicles, reduces CL, and induces ovarian fibrosis	Ovarian morphological changes are positively correlated with serum iron levels	[71]
Iron (elemental, excess)	Subfertile cows	\	1. Iron overload correlates with significantly increased follicular atresia rates and ovarian collagen deposition (*p* < 0.05) 2. Disrupts pituitary–ovarian axis function, accelerates follicular atresia (causal association unconfirmed in cattle)	1. Estrogen regulates iron homeostasis via hepcidin; iron overload disrupts this balance 2. Iron overload potentially damages ovarian structure via pro-fibrotic pathways (conceptual reference only)	[74,75,76]
Chelated iron	Multiparous Jersey cows (210 ± 18 DIM, 25 kg/d milk yield, 4 ± 0.6 months gestation)	600 mg/head/d (30 mg/kg DM, iron overload/toxic dose)	1. Elevates serum and milk iron levels, induces oxidative stress, immunosuppression and potential liver damage 2. Reduces final 10-day milk yield, increases disease incidence (7 mastitis, 2 intestinal paresis) 3. Disrupts glucose metabolism and intestinal microbiota	Iron overload triggers systemic metabolic dysfunction and reproductive tissue oxidative damage	[77]

#### 6.2.2. Interaction Between Iron Metabolism and Reproductive Hormones

The following mechanistic inferences are primarily derived from non-ruminant models, and the specific pathways in cattle require confirmation through cattle-specific research. During the bovine reproductive cycle, E2 regulates iron homeostasis by modulating hepcidin. Iron overload disrupts this balance, leading to pituitary–ovarian axis dysfunction manifested as gonadotropin secretion disorders and accelerated follicular atresia. In subfertile cow populations, serum iron concentration showed significant positive correlations (*p* < 0.05) with follicular atresia rates and ovarian collagen deposition, suggesting that iron overload directly damages ovarian structure via pro-fibrotic pathways [74,75,76].

#### 6.2.3. Effects of Iron on Oxidative Stress and Reproductive Tissue Damage

The ferroptosis pathway plays a pivotal role in premature ovarian failure. Excess iron induces ferroptosis in theca cells via the GPX4 pathway, leading to diminished ovarian reserve. Granulosa cells (GCs) in bovine ovaries are critical for follicular development and steroid hormone synthesis; their proliferation and functional stability directly influence estrus, ovulation, and reproductive performance [73]. Ferroptosis-related pathways and genes induced by iron overload may regulate granulosa cell proliferation and function; Ferrous ion can directly inhibit granulosa cell proliferation and arrest the cell cycle by modulating ROS-mediated p38MAPK/p53/p21 pathways [73]. However, no studies have yet investigated these mechanisms in cattle.

Iron overload exerts detrimental impacts on the health and productivity of dairy cows, and this study was conducted to verify such effects in multiparous Jersey cows. Twenty-four multiparous Jersey cows (210 ± 18 days in milk, 25 kg daily milk yield, 4 ± 0.6 months of gestation) were assigned to two groups (*n* = 12 each): a control group with no iron supplementation and a treatment group administered 600 mg chelated iron per animal daily (30 mg/kg dry matter). Sampling on days 1, 16, 29 and 42 revealed that iron-supplemented cows had elevated serum and milk iron levels, but also exhibited immunosuppression-related hematological perturbations, altered glucose metabolism, potential liver damage, enhanced oxidative stress, intestinal microbiota dysbiosis, reduced milk yield in the final 10 days, and high disease incidence (7 mastitis cases, 2 intestinal paresis cases). This dose of chelated iron is therefore not recommended, as it compromises dairy cow health and productivity [77].

## 7. Future Directions

Existing studies have systematically clarified the regulatory roles of five essential trace elements (copper, selenium, zinc, cobalt, iron) in female cattle reproductive function. Copper modulates steroidogenesis via the AKT/WNT and FSHR/CYP19A1 pathways to improve oocyte maturation and pregnancy rates. Selenium activates the Nrf2 pathway to enhance ovarian and uterine antioxidant defense, supporting luteal function and embryonic development; zinc acts as a cofactor for steroidogenic enzymes (e.g., 3β-HSD) and strengthens immunity/antioxidant capacity to stabilize the reproductive hormone environment. Cobalt regulates the HPO axis via vitamin B_12_ to optimize hormonal balance and nitrogen metabolism. Iron exhibits concentration-dependent dual effects (adequate levels support hormone secretion and estrous cycle stability, excess induces ovarian damage and ferroptosis)—all elements are highly dose-sensitive, deficiency or excess disrupts reproductive physiology, and inter-element synergism/antagonism further influences bioavailability and outcomes.

However, several critical knowledge gaps remain. The specific requirements for these trace elements across different reproductive stages in female cattle, such as estrus, pregnancy, and lactation, have not been systematically investigated. The interactions between trace elements and associated functional proteins in regulating reproductive performance also lack a comprehensive analysis. Moreover, the molecular mechanisms and key targets underlying each element’s influence on fertility remain unclear, and studies addressing bioavailability, metabolic pathways, and efficacy variations among different element sources (e.g., nanomaterials, organic, and inorganic compounds) are still limited. Future research should aim to bridge these gaps by conducting comparative analyses of elements derived from diverse sources to identify high-efficacy supplements, elucidating the molecular targets and protein interaction networks of each element, and integrating reproductive stage-specific physiological characteristics and nutritional requirements to establish scientifically grounded, precise, and personalized nutritional strategies. Such efforts will provide a robust theoretical foundation and practical guidance for enhancing reproductive efficiency in female cattle, reducing metabolism-related reproductive disorders, and promoting the sustainable and healthy development of animal husbandry.

In addition, proteomics enables the identification of mineral-dependent functional proteins, such as zinc-finger transcription factors, copper-containing superoxide dismutase, and iron-associated cytochrome P450 enzymes, clarifying how minerals influence protein activity and stability to mediate reproductive processes. Metabolomics, in turn, links mineral status to changes in key metabolites (e.g., progesterone, glutathione, malondialdehyde), constructing a comprehensive “mineral–gene–metabolite” regulatory axis that bridges molecular mechanisms with physiological outcomes. Integrating multi-omics data will further uncover novel molecular targets and signaling cascades, such as cobalt’s vitamin B12-dependent regulation of the hypothalamic–pituitary–ovarian axis, providing a more precise theoretical basis for tailored mineral supplementation strategies.

This review consolidates key findings on how trace minerals (copper, selenium, zinc, cobalt, and iron) regulate reproductive efficiency in female cattle through specific mechanisms, such as enhancing antioxidant defense, hormone synthesis, and cellular signaling. These insights directly support the research objective of elucidating mineral–reproduction relationships by providing a mechanistic basis for optimizing supplementation strategies, thereby improving pregnancy rates, reducing embryonic loss, and promoting sustainable cattle production.

The findings underscore the critical role of balanced mineral nutrition in advancing bovine reproductive health, offering practical guidelines for enhancing fertility and supporting global food security through evidence-based management.

## Figures and Tables

**Table 1:** Detailed literature search strategy covering 1992–2025.

Search Category (Specific Topic)	Keywords/Search Query String
General Scope & Review Framework	(trace mineral* OR micronutrient*) AND (cattle OR dairy cow* OR beef cow*) AND (reproduct* OR fertility)
Copper Deficiency & Reproduction	(copper deficiency OR hypocurremia) AND (cattle OR bovine) AND (conception rate OR embryonic loss OR estrus delay)
Selenium, Antioxidants & Endometrium	(selenium OR Se) AND (cattle OR bovine) AND (endometrium OR endometrial cell*) AND (oxidativ* stress OR antioxidant)
Zinc, Immunity & Postpartum Health	(zinc OR Zn) AND (cattle OR bovine) AND (immune* OR postpartum OR metritis) AND (reproduct* recovery)
Cobalt/Vitamin B12 & Hormonal Regulation	(cobalt deficiency OR vitamin B12 OR cobalamin) AND (cattle OR ruminant*) AND (progesterone OR estradiol OR HPO axis)
Iron Overload & Ovarian Toxicity	(iron overload OR excess iron) AND (cattle OR bovine OR ovary) AND (ferroptosis OR follicular atresia OR oxidativ* damage)
Organic vs. Inorganic Mineral Sources	(organic mineral* OR chelated mineral* OR amino acid complex*) AND (inorganic mineral* OR sulfate OR oxide) AND (cattle OR bovine) AND (bioavailability OR reproduct* performance)

The asterisk “*” in the table is used as a wildcard character.

**Table 2:** Copper: Effects and mechanisms on reproductive function in cattle.

Resource	Animal/Cell	Upper Limit Value	Major Effects	Mechanism	Reference
Copper	Angus Beef Cattle; Dairy Cows	\	1. Copper deficiency (<30 µg/dL) linked to reduced conception, delayed estrus, early embryonic loss 2. Supplementation improves pregnancy rate (beef cattle); reduces anovulatory P4-free follicles & plasma E2 (dairy cows)	Acts as cofactor in antioxidation, hormone synthesis and cell signaling	[12]
Copper sulfate (CuSO_4_)	Yak COC		50 µM (supplemented in maturation media) enhances oocyte developmental competence, promotes nuclear/cytoplasmic maturation, reduces CCs apoptosis	Improves cumulus–oocyte gap junction communication, facilitates GSH transfer, maintains redox homeostasis	[15,16]
	Yak Granulosa cells	0.65 mM	Concentration-dependent E2 regulation: 0.25 mM increases E2; 0.65 mM inhibits E2	1. Activates AKT/WNT pathways, upregulates CYP19A1, StAR, CYP11A1, 3β-HSD, 17β-HSD 2. 0.65 mM suppresses FSHR/CYP19A1 pathway	[17,18]
Copper–zinc complex	Nellore Heifers	\	Enhances luteal size and plasma GPx concentration; improves estrus expression (low BCS)	1. Synergizes with zinc to support luteal growth and antioxidative protection 2. Regulates hormone synthesis and antioxidative defense	[17]
Copper–amino acid complex	Angus; Simmental Heifers		Supplementation reduces embryonic mortality; increases hepatic cytochrome oxidase and PAGs levels	Modulates hepatic metabolism and pregnancy-related protein expression	[20]
Organic-complexed copper; Hydroxychloride copper	Angus Cows		Late gestation supplementation promotes postpartum body condition recovery (late gestation supplementation)	Supports maternal metabolic homeostasis and postpartum recovery	[21]

## Data Availability

No new data were created or analyzed in this study. Data sharing is not applicable to this article.

## Abbreviations

The following abbreviations are used in this manuscript:

COC	Cumulus–Oocyte Complex
GPx	Glutathione peroxidase
StAR	Steroidogenic acute regulatory protein
CYP19A1	Cytochrome P450 family 19 subfamily A member 1
17β-HSD	17β-hydroxysteroid dehydrogenase
Nrf2	Nuclear factor erythroid 2-related factor 2
HPO	Hypothalamic–pituitary–ovarian
HPG	Hypothalamic–pituitary–gonadal
GnRH	Gonadotropin-releasing hormone
LH	Luteinizing hormone
FSH	Follicle-stimulating hormone
LPS	Lipopolysaccharide
CTGF	Connective tissue growth factor
TGF-β	Transforming growth factor-β
ISGs	Interferon-stimulated genes
SOD	Superoxide dismutase
CAT	Catalase
GSH	Glutathione
MDA	Malondialdehyde
T-AOC	Total antioxidant capacity
CL	Corpus luteum
BESCs	Bovine endometrial stromal cells
BCS	Body condition score
ROS	Reactive oxygen species
TP	Total protein
OCM	A combination of folic acid, methionine, and choline
CP	Crude protein
BGL	Bovine genital leptospirosis
IGF-1	Insulin-like growth factor-1
CCs	Cumulus cells
IVM	In vitro maturation
DM	Dry matter
LDLR	Low-density lipoprotein receptor
ER	Estrogen receptor
Arg	Arginine
EGF	Epidermal growth factor
CLBF	Corpus luteal blood flow
E2	Estradiolum
P4	progesterone
HSD	Hydroxysteroid dehydrogenases
CTM	Critical trace minerals
PAGs	Pregnancy-associated glycoproteins
SCC	Somatic cell counts
BGCs	Bovine granulosa cells
AAC	Amino acid complexes
GCs	Granulosa cells
